# The effect of cognitive load, ego depletion, induction and time restriction on moral judgments about sacrificial dilemmas: a meta-analysis

**DOI:** 10.3389/fpsyg.2024.1388966

**Published:** 2024-05-02

**Authors:** Paul Rehren

**Affiliations:** Ethics Institute, Department of Philosophy and Religious Studies, Utrecht University, Utrecht, Netherlands

**Keywords:** moral judgment, dual-process model, sacrificial dilemmas, cognitive-processing manipulations, meta-analysis

## Abstract

Greene's influential dual-process model of moral cognition (mDPM) proposes that when people engage in Type 2 processing, they tend to make consequentialist moral judgments. One important source of empirical support for this claim comes from studies that ask participants to make moral judgments while experimentally manipulating Type 2 processing. This paper presents a meta-analysis of the published psychological literature on the effect of four standard cognitive-processing manipulations (cognitive load; ego depletion; induction; time restriction) on moral judgments about sacrificial moral dilemmas [*n* = 44; *k* = 68; total *N* = 14, 003; *M*(*N*) = 194.5]. The overall pooled effect was in the direction predicted by the mDPM, but did not reach statistical significance. Restricting the dataset to effect sizes from (high-conflict) personal sacrificial dilemmas (a type of sacrificial dilemma that is often argued to be best suited for tests of the mDPM) also did not yield a significant pooled effect. The same was true for a meta-analysis of the subset of studies that allowed for analysis using the process dissociation approach [*n* = 8; *k* = 12; total *N* = 2, 577; *M*(*N*) = 214.8]. I argue that these results undermine one important line of evidence for the mDPM and discuss a series of potential objections against this conclusion.

## 1 Introduction

Greene's *dual-process model of moral cognition* (mDPM^1^; Greene et al., 2001; Greene, 2008, 2014) is one of the most well-known and influential models in moral psychology. Like other dual-process models, the mDPM makes the core assumption that “cognitive tasks evoke two forms of processing that contribute to observed behavior” (Evans and Stanovich, 2013, p. 225). These two forms of processing go by a variety of different names; here, I will refer to them as *Type 1* and *Type 2* processing. Ways to spell out the distinction between Type 1 and Type 2 processing abound (see e.g., Evans, 2008;

Evans and Stanovich, 2013). According to Greene et al. (2008, p. 40–1) and Greene (2014, p. 698–9), Type 1 processes are quick, effortless and unconscious, while Type 2 processes are slower, require effort and operate at the level of consciousness.

The central idea of the mDPM is that Type 1 and Type 2 processing tend to produce different kinds of moral judgment. When people engage in Type 1 processing, then they typically make *deontological* moral judgments—moral judgments that are “naturally justified in deontological terms (in terms of rights, duties, etc.) and that are more difficult to justify in consequentialist terms.”^2^ In contrast, when people engage in Type 2 processing, then they typically make *consequentialist* moral judgments—moral judgments that are “naturally justified in consequentialist terms (i.e., by impartial cost-benefit reasoning) and that are more difficult to justify in deontological terms because they conflict with our sense of people's rights, duties, and so on” (both, Greene, 2014, p. 699).

One way to illustrate the mDPM is with two classic *sacrificial moral dilemmas*. Sacrificial dilemmas are a type of moral dilemma; they involve situations where the agent can either do nothing or intervene (see e.g., Kahane et al., 2018, p. 132; Klenk, 2022, p. 593–594). If the agent does nothing, this will result in harm to or the death of one or more individuals. If the agent does intervene, she will save some or all of these individuals; however, the intervention will also cause harm, though less harm than would result from the agent doing nothing (e.g., a smaller number of individuals get killed). In one classic example (*Switch*), an out-of-control trolley is speeding toward five people. The only way to save them is to divert the trolley onto a separate track by hitting a switch. However, there is a sixth person on this other track who will then be run over and killed by the trolley if it is diverted (Foot, 1967). Another classic example (*Footbridge*) again features an out-of-control trolley; this time, however, the only way to save the five people is to push a sixth person off a footbridge and into the path of the trolley (Thomson, 1976). People tend to give the consequentialist response to Switch (that is, they endorse hitting the switch) but the deontological response to Footbridge (that is, they do not endorse pushing the person off the footbridge; e.g., Hauser et al., 2007; Awad et al., 2020).

To explain this finding, Greene et al. (2001, 2004) have argued that when most people consider Footbridge, this causes them to have a strong Type 1 response, one that only rarely gets overridden by subsequent Type 2 processing. Therefore, most people end up endorsing the deontological option in Footbridge. In contrast, considering Switch only causes a weak Type 1 response or no immediate Type 1 response at all, and so more people choose the consequentialist option in Switch than in Footbridge.

One important source of evidence for the mDPM (e.g., Kahane, 2012, p. 524–526; Greene, 2014, p. 700–705; Guglielmo, 2015, p. 10–11) comes from studies that investigate the effect on moral judgments of experimental manipulations designed to either encourage participants to engage in Type 2 processing or to inhibit their ability to engage in Type 2 processing. Common methods to encourage Type 2 processing include direct instruction and time delays; common methods to inhibit Type 2 processing include cognitive load, time pressure and direction instruction (see Horstmann et al., 2010; Isler and Yilmaz, 2023). Most of these studies feature moral judgments about sacrificial moral dilemmas. The mDPM predicts that when participants are encouraged to engage in Type 2 processing, this will increase consequentialist responding, while inhibiting Type 2 processing will reduce consequentialist responding. However, previous overviews of the evidence have concluded that this body of studies only provides “inconsistent support” (Patil et al., 2021, p. 445) for the mDPM: Some studies have borne out the mDPM's predictions, but others have not. To what extent this supports the mDPM overall, then—if it does indeed support it at all—is to date unknown.

In this paper, I aim to change this. To this end, I present a meta-analysis of published English-language psychological studies with adult participants investigating the effect of four standard cognitive-processing manipulations (cognitive load; ego depletion; induction; time restriction) on moral judgments about sacrificial moral dilemmas. The meta-analysis includes a total of 44 articles, reporting results from 68 individual studies [total *N* = 14, 003; *M*(*N*) = 194.5]. Section 2 provides details about my inclusion criteria and the literature search. Section 3 then presents the meta-analysis, along with an analysis of the potential presence of publication bias and a series meta-regressions (manipulation type; experimental design; sample location; sample type; dilemma type; manipulation check; type of literature). To end, I discuss the implications of my results for the mDPM.

## 2 Method

### 2.1 Inclusion criteria

I searched for and included published English-language studies with adult participants investigating the effect of four standard cognitive-processing manipulations (cognitive load; ego depletion; induction; time restriction) on moral judgments about sacrificial moral dilemmas. I defined moral judgments as judgments about what one should or should not do in a situation, where this includes judgments about an action's or a decision's wrongness, permissibility, appropriateness and acceptability. This means that studies which measured moral behavior instead of moral judgment (e.g., offers in an economic game), asked participants to make a hypothetical choice (e.g., Would you push the button?), asked participants to indicate a preference (e.g., Which outcome would you prefer?) or asked participants to make a judgment about themselves, are beyond the scope of this review (see McDonald et al., 2021, p. 3). In addition, I excluded studies if they did not report original empirical data (e.g., review articles), only reported data from non-adult participants, had been published in a language other than English or had been retracted.

Various approaches have been tried to encourage and inhibit Type 2 processing (for overviews, see Horstmann et al., 2010; Isler and Yilmaz, 2023). Like two other recent meta-analyses (Rand, 2016; Kvarven et al., 2020), I focused on four standard cognitive-processing manipulations: cognitive load, ego depletion, induction and time restriction.

*Cognitive load* studies ask some participants to engage in a difficult cognitive task at the same time that they are making moral judgments. There are a variety of these tasks for researchers to choose from; common examples include tasks that tax participants' memory (e.g., memorize a complicated pattern of dots on a 4 × 4 grid) and tasks that require participants to pay attention to an additional stimulus (e.g., listen to a list of numbers and count how many prime numbers there are). The responses of participants under cognitive load are then compared to the responses of participants who engaged in an easier version of the load task (e.g., memorize a simple pattern of dots on a 3 × 3 grid) or no additional task, with the idea being that participants under cognitive load have fewer cognitive resources available for the moral judgment task and so will be inhibited from engaging in Type 2 processing while completing the task (Gilbert et al., 1988).

*Ego depletion* studies also aim to limit the amount of cognitive resources that participants have available to them while rendering moral judgments. They achieve this either by asking participants to complete a taxing cognitive task prior to the moral judgment task, or through mental or physical exertion (e.g., sleep deprivation; stress; hunger). Compared to participants who have completed a milder version of the depletion task or no task at all, depleted participants are supposed to be more mentally fatigued and so are less able or willing to engage in Type 2 processing (Hagger et al., 2010; but see Carter et al., 2019).

*Induction* studies encourage or discourage participants to engage in Type 2 processing while making moral judgments. One common way to achieve this it to give participants explicit instructions (e.g., think carefully and logically before making your judgment; think about how you would justify your judgment). Other induction studies have relied on unconscious primes. Many different primes have been tried, including memory recall (e.g., asking participants to think back to a time when reflection lead to a favorable outcome), tasks like the Cognitive Reflection Test and the Berlin Numeracy Test (Cokely et al., 2012), and deliberative mindset primes.^3^

Finally, *time restriction* studies either limit the amount of time that participants have to make their moral judgment, or only allow participants to make their judgment after a time delay. Time-pressured and time-delayed responses are then compared with each other, or with the responses from participants whose response time was unconstrained. Time pressure makes it less that participants engage in Type 2 processing because they lack the time to do so. Conversely, time delays are thought to make it more likely that participants will engage in Type 2 processing to make their judgment (Wright, 1974).

### 2.2 Literature search

**Figure 1:**
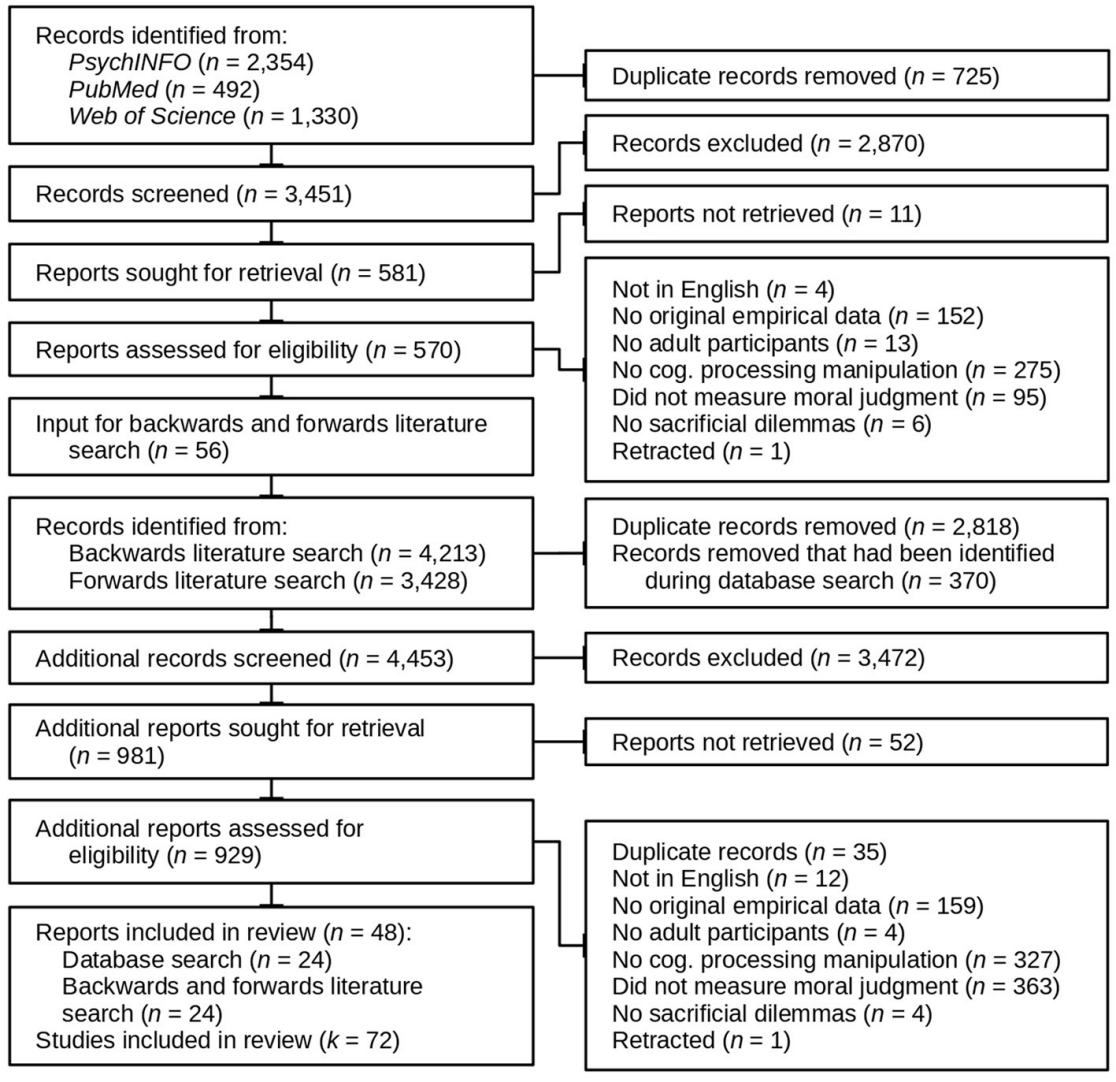
Flow diagram illustrating the literature search and the different inclusion and exclusion stages (Page et al., 2021a).

The literature search was carried out in two steps (for details, see Figure 1). The first step was a database search; in the second step, I carried out backward and forward literature searches on all articles identified in the first step (plus some additional articles, see below). For the database search, I searched for relevant studies within *PubMed, PsycINFO* and *Web of Science*, using an intentionally broad combination of search terms like “reflection”, “deliberation”, “moral judgment” and “moral decision-making” (for details, see: osf.io/h2fcp/). This search yielded 3,451 unique records, which I then screened based on their titles. Finally, I assessed the full texts of the remaining records for eligibility. This left 24 peer reviewed articles to be included in the review.

The second step consisted of a forward and backward literature search (pre-registered at: osf.io/gysuj/). These searches were carried out using Google Scholar, queried through Publish or Perish (Harzing, 2021). The input to this search were the 24 articles previously identified through database search, plus 26 additional articles that investigated the relationship between moral judgment and the tendency of participants to engage in intuitive and reflective processing (for an overview, see Patil et al., 2021) and six additional articles that investigated the relationship between moral judgment and cognitive processing with materials other than sacrificial dilemmas. These additional articles had also been identified in the initial database search. Forward and backward literature search resulted in an additional 4,418 unique records, which after screening and assessment for eligibility contributed an additional 24 articles.

All in all, I identified 48 articles that matched my inclusion criteria, which reported results from 72 individual studies.

### 2.3 Data collection

The majority of studies reported continuous outcomes. Therefore, I chose to use the bias-corrected standardized mean difference as the common effect size for the meta-analysis (Hedges' *g*_s_ for between-subject studies, Hedges' *g*_av_ for within-subject studies—I will drop the subscripts for the rest of the paper; Lakens, 2013). The remaining studies reported binary outcomes. Since these two sets of studies were otherwise very similar, I combined both in a single meta-analysis (see Borenstein et al., 2009, chap. 7). The meta-analysis included both between-subject and within-subject studies. To make these two sets of studies comparable, I converted all within-subject effect sizes to between-subject effect sizes (Morris and DeShon, 2002, Eq. 11). I coded all effect sizes so that positive values of *g* indicated results in line with the expectations of the mDPM: participants who were encouraged to engage in Type 2 processing made more consequentialist moral judgments than participants in a control condition or participants whose ability to engage in Type 2 processing was inhibited, while participants whose ability to engage in Type 2 processing was inhibited made less consequentialist moral judgments than participants in a control condition.

Most studies reported sufficient statistical information to calculate *g*. For studies where this was not the case, I contacted the corresponding author(s) and requested the necessary statistical information. In the end, I was able to calculate *g* for all but four studies. None of the studies I was not able to include reported a significant effect of their cognitive-processing manipulation on participants' moral judgments.^4^ In the end, the meta-analysis included 44 articles, reporting results from 68 individual studies [total *N* = 14, 003; *M*(*N*) = 194.5]. Table 1 shows a detailed overview of these studies.

**Table 1:** Detailed information about all studies included in the meta-analysis; “—” signifies that a piece of information was not reported in the article in question.

			**Type 2 processing manipulation**				**Sample**		**Manipulation check**				
**Study**	**Design**	***N***	**Type**	**Encouraged**	**Control**	**Inhibited**	**Location**	**Type**	**Type**	**Status**	**Personal (%)**	**PD**	**Published**
Bago and De Neys (2019), Study 4	Within	101	Induction; load; time	Instructions to consider the scenario carefully and to take one's time		Dot memorization task; time pressure	Europe	Online		None	50.0	Yes	Yes
Białek and De Neys (2017), Study 2	Between	141	Load		Control	Dot memorization task	North America	Online		None	0.0	Yes	Yes
Białek and De Neys (2017), Study 3	Between	151	Load		Control	Dot memorization task	North America	Online		None	0.0	Yes	Yes
Cellini et al. (2017)	Between	37	Depletion		Control	Sleep deprivation	Europe	Students	Compared self-reported sleepiness and fatigue between conditions	Success	50.0	No	Yes
Chaturapanich and Chaiwutikornwanich (2015), Study 1	Between	360	Induction; time	Instructions to think carefully		Time pressure	Other	Students		None	0.0	No	Yes
Chaturapanich and Chaiwutikornwanich (2015), Study 2	Between	360	Induction; time	Instructions to think carefully		Time pressure	Other	Students		None	0.0	No	Yes
Conway and Gawronski (2013), Study 2	Between	56	Load		Control	String memorization task	North America	Students		None	50.0	Yes	Yes
Cova et al. (2018), 2008—most cited	Within	85	Load		Control	Digit attention task	Europe	Other		None	100.0	No	Yes
Cova et al. (2018), 2012—most cited	Between	298	Induction	Cognitive Reflection Test	Control		Europe	Online		None	100.0	No	Yes
Cummins and Cummins (2012), Study 1	Between	182	Time		Control	Time pressure	North America	Students	Compared response times between conditions	Success	64.7	No	Yes
Cummins and Cummins (2012), Study 2	Within	65	Time		Control	Time pressure	North America	Students	Compared response times between conditions	Success	64.7	No	Yes
Doerflinger and Gollwitzer (2020), Study 1	Between	204	Induction	Deliberative mindset prime	Control		—	Online		None	100.0	No	Yes
Gamez-Djokic and Molden (2016), Study 7	Within	51	Load		Control	String memorization task	North America	Students		None	50.0	No	Yes
Gamez-Djokic and Molden (2016), Study 8	Within	144	Induction	Instructions to think carefully and logically and to take one's time	Control		—	Online		None	50.0	No	Yes
Gamez-Djokic and Molden (2016), Study 9	Within	143	Induction	Instructions to think carefully and logically and to take one's time	Control		—	Online		None	50.0	No	Yes
Gawronski et al. (2017), Study 2a	Between	194	Load		Control	String memorization task	—	Online	Compared memorization accuracy between conditions	Success	66.7	Yes	Yes
Gawronski et al. (2017), Study 2b	Between	194	Load		Control	String memorization task	—	Online	Compared memorization accuracy between conditions	Success	66.7	Yes	Yes
Greene et al. (2008)	Between	82	Load		Control	Digit attention task	North America	Students		None	100.0	No	Yes
Gürçay and Baron (2017), Study 1	Within	64	Induction	Instructions to make reflective judgments		Instructions to make intuitive judgments	—	Online	Compared response times between conditions	Success	50.0	No	Yes
Gürçay and Baron (2017), Study 2	Between	55	Induction	Instructions to make reflective judgments		Instructions to make intuitive judgments	—	Online	Compared response times between conditions	Success	50.0	No	Yes
Gürçay and Baron (2017), Study 3	Between	194	Induction; time	Instructions to make reflective judgments; time delay		Instructions to make intuitive judgments; time pressure	—	Online	Compared response times between conditions	Success	47.8	No	Yes
Gürçay and Baron (2017), Study 3	Within	85	Induction; time	Instructions to make reflective judgments; time delay		Instructions to make intuitive judgments; time pressure	—	Online	Compared response times between conditions	Failed	47.8	No	Yes
Ham and van den Bos (2010)	Between	100.0	Induction; time	Instructions to deliberate; time delay	Control		Europe	Students		None	100.0	No	Yes
Hashimoto et al. (2022)	Within	119	Time		Control	Time pressure	Other	Students		None	0.0	No	Yes
Houston (2010), Study 1	Between	189	Load		Control	String memorization task	North America	Other		None	100.0	No	No
Houston (2010), Study 2	Between	292	Load		Control	String memorization task	North America	Other		None	100.0	No	No
Houston (2010), Study 3	Between	106	Load		Control	String memorization task	North America	Other		None	100.0	No	No
Houston (2010), Study 4	Between	99	Time		Control	Time pressure	North America	Other		None	100.0	No	No
Killgore et al. (2007)	Within	26	Depletion		Control	Sleep deprivation	North America	Other		None	50.0	No	Yes
Kroneisen and Steghaus (2021), Study 1	Between	199	Time	Time delay		Time pressure	Europe	Other	Compared response times between conditions	Success	75.0	Yes	Yes
Kroneisen and Steghaus (2021), Study 2	Between	168	Induction	Instructions to take their time and deliberate		Instructions to answer quickly and intuitively	Europe	Other	Compared response times between conditions	Failed	66.7	Yes	Yes
Körner and Volk (2014), Study 1	Between	112	Time	Time delay		Time pressure	—	Online		None	100.0	No	Yes
Li et al. (2018), Study 2	Between	43	Induction; load	Instructions to deliberate		Letter memorization task; instructions to make one's decision based on feeling	Other	Students	Compared memorization accuracy between conditions	Success	66.7	Yes	Yes
Li et al. (2021)	Between	78	Depletion		Control	Trier Social Stress Test	Other	Students	Compared self-reported stress and heart rate between conditions	Success	66.7	Yes	Yes
Liu and Liao (2021), Study 3	Between	180	Induction; load	Instructions to think about the reasons for one's decision		Letter memorization task	Other	Students		None	100.0	No	Yes
Lyrintzis (2017), Study 1c	Between	104	Induction	Cognitive Reflection Test	Control		North America	Online		None	100.0	No	No
McPhetres et al. (2018), Study 1	Between	220	Time		Control	Time pressure	—	Other		None	50.0	Yes	Yes
McPhetres et al. (2018), Study 2	Between	186	Load		Control	String memorization task	North America	Students		None	50.0	Yes	Yes
McPhetres et al. (2018), Study 3	Between	801	Load		Control	String memorization task	North America	Online		None	50.0	Yes	Yes
Paxton et al. (2012), Study 1a	Between	92	Induction	Cognitive Reflection Test	Control		—	Online		None	100.0	No	Yes
Paxton et al. (2012), Study 1b	Between	72	Induction	Cognitive Reflection Test	Control		North America	Students		None	100.0	No	Yes
Paxton et al. (2014)	Between	17	Induction	Cognitive Reflection Test	Control		—	Online		None	100.0	No	Yes
Reid (2018)	Between	166	Load		Control	String memorization task	Other	Other	Compared self-reported task difficulty between conditions	Success	62.5	No	No
Rosas and Aguilar-Pardo (2020), Study 1	Between	344	Induction; time	Instructions to consider the scenario carefully and to take one's time; time delay		Instructions to think fast; time pressure	—	Online		None	100.0	No	Yes
Rosas and Aguilar-Pardo (2020), Study 2	Between	709	Induction; time	Instructions to consider the scenario carefully and to take one's time; time delay		Instructions to think fast; time pressure	—	Online		None	100.0	No	Yes
Rosas and Aguilar-Pardo (2020), Study 3	Between	657	Induction; time	Instructions to consider the scenario carefully and to take one's time; time delay		Instructions to think fast; time pressure	—	Online		None	100.0	No	Yes
Rosas and Aguilar-Pardo (2020), Study 4	Between	554	Induction; time	Instructions to consider the scenario carefully and to take one's time; time delay		Instructions to think fast; time pressure	—	Online		None	100.0	No	Yes
Rosas et al. (2019), Study 3	Between	284	Load		Control	Dot memorization task	Other	Online	Compared response times between conditions	Success	100.0	No	Yes
Schwitzgebel and Cushman (2015)	Between	2686	Induction; time	Instructions to consider the scenario carefully and to take one's time; time delay	Control		North America	Other	Compared response times between conditions	Success	66.6	No	Yes
Simpson (2021)	Between	182	Induction		Control	Cognitive Reflection Test	North America	Students		None	100.0	No	No
Spears et al. (2021), Study 1a—BNT	Between	83	Induction	Berlin Numeracy Test with feedback	Control		Europe	Students	Compared rates of correct answers on the Berlin Numeracy Test before and after feedback	Success	100.0	No	Yes
Spears et al. (2021), Study 1a—CRT	Between	138	Induction	Cognitive Reflection Test with feedback	Control		Europe	Students	Compared rates of correct answers on the Cognitive Reflection Test before and after feedback	Success	100.0	No	Yes
Spears et al. (2021), Study 1b—BNT	Between	99	Induction	Berlin Numeracy Test with feedback	Control		Europe	Online	Compared rates of correct answers on the Berlin Numeracy Test before and after feedback	Success	100.0	No	Yes
Spears et al. (2021), Study 1b—CRT	Between	86	Induction	Cognitive Reflection Test with feedback	Control		Europe	Online	Compared rates of correct answers on the Cognitive Reflection Test before and after feedback	Success	100.0	No	Yes
Starcke et al. (2012)	Between	50	Depletion		Control	Stress induction task	Europe	Students	Compared heart rate between conditions	Success	50.0	No	Yes
Suter and Hertwig (2011), Study 1	Between	67	Time	Time delay		Time pressure	Europe	Students	Compared response times between conditions	Success	50.0	No	Yes
Suter and Hertwig (2011), Study 2	Between	80	Induction	Instructions to take one's time to deliberate		Instructions to answer quickly and intuitively	Europe	Students	Compared response times between conditions	Success	50.0	No	Yes
Swann et al. (2014), Study 5	Between	436	Time		Control	Time pressure	Europe	Students		None	0.0	No	Yes
Timmons and Byrne (2019), Study 1	Between	196	Depletion		Control	Online depletion task	Other	Online	Compared self-reported task difficulty between conditions	Success	50.0	No	Yes
Timmons and Byrne (2019), Study 2	Between	187	Depletion		Control	Online depletion task	Other	Online	Compared self-reported task difficulty between conditions	Success	50.0	No	Yes
Tinghög et al. (2016), Study 1	Between	1100	Time	Time delay		Time pressure	Other	Other	Compared performance on Jellybean task between conditions	Success	50.0	No	Yes
Tinghög et al. (2016), Study 2	Between	311	Load		Control	Digit memorization task/attention depletion task	Europe	Students	Compared self-reports of how much energy the load task took between conditions	Success	50.0	No	Yes
Trémolière and Bonnefon (2014), Study 1	Between	213	Load		Control	Dot memorization task	Europe	Online		None	100.0	No	Yes
Trémolière and Bonnefon (2014), Study 2	Between	123	Time		Control	Time pressure	Europe	Online		None	100.0	No	Yes
Trémolière and Bonnefon (2014), Study 3	Between	204	Time		Control	Time pressure	Europe	Online		None	75.0	No	Yes
Trémolière et al. (2012), Study 2	Between	110	Load		Control	Dot memorization task	Europe	Students		None	100.0	No	Yes
Van't Veer and Sleegers (2019)	Between	185	Depletion		Control	Anticipatory stress task	Europe	Students	Compared self-reported stress between conditions	Success	0.0	No	Yes
Vega et al. (2021), Study 1	Between	59	Induction		Control	Instructions to give the first answer that comes to mind	—	Online	Compared response times between conditions	Success	100.0	No	Yes
Vega et al. (2021), Study 1	Within	30	Induction	Instructions to consider the scenario carefully and to take one's time		Instructions to give the first answer that comes to mind	—	Online	Compared response times between conditions	Success	100.0	No	Yes
Vega et al. (2021), Study 2	Within	119	Induction	Instructions to consider the scenario carefully and to take one's time		Instructions to give the first answer that comes to mind	—	Online	Compared response times between conditions	Success	50.0	No	Yes
Weippert et al. (2018)	Between	32	Depletion		Control	High intensity ergometer cycling session	Europe	Other	Compared cortisol levels and self-reported effort, pain, fatigue, mood, wakefulness and arousal between conditions	Success	50.0	No	Yes
Youssef et al. (2012)	Between	65	Depletion		Control	Trier Social Stress Test	Other	Students	Compared cortisol levels between conditions	Success	50.0	No	Yes
Zhao et al. (2016)	Between	159	Depletion		Control	Anxiety induction task	North America	Students	Compared self-reported anxiety between conditions	Success	50.0	No	Yes
Zheng (2018), Study 6	Between	137	Induction	Instructions to consider the scenario carefully and to take one's time	Control		Europe	Students		None	100.0	No	No

## 3 Results

### 3.1 Point estimate

**Figure 2:**
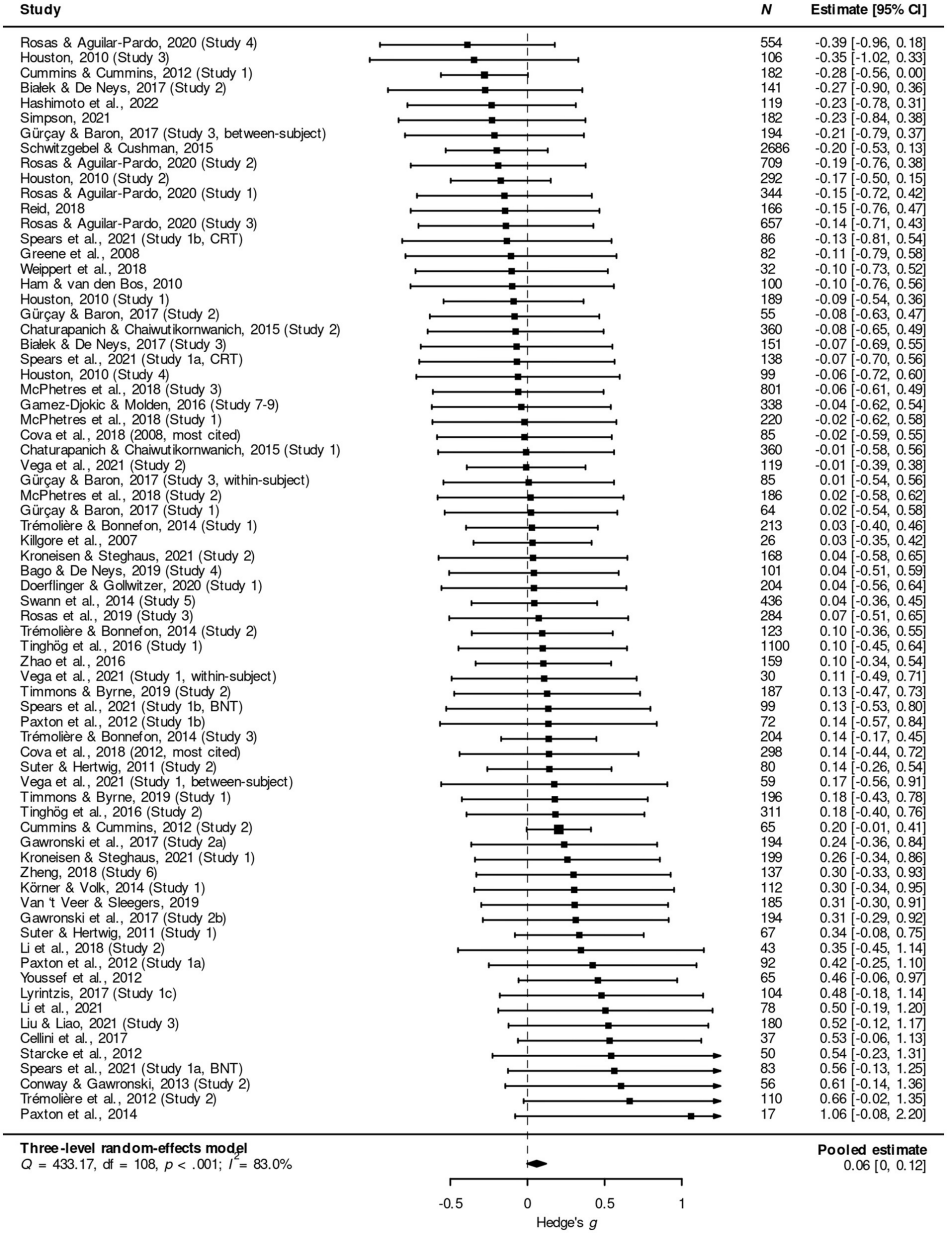
Forest plot showing the meta-analytic effect of four standard cognitive-processing manipulations on moral judgments about acts or decisions in sacrificial moral dilemmas. Positive values of *g* indicate results in line with the mDPM.

All analyses were carried out in *R* (R Core Team, 2023).^5^ To calculate a point estimate and 95% confidence interval for the combined effect of cognitive load, ego depletion, induction and time restrictions on moral judgments about sacrificial dilemmas, I used a three-level random-effects model, with effect sizes nested within studies. This was done to account for the fact that some studies contributed multiple effect sizes (see Harrer et al., 2021, chap. 10). While there was a small (Cohen, 1988) positive pooled effect, it did not reach statistical significance: *g* = 0.06, 95% CI = (0.00, 0.12), *p* = 0.057. Figure 2 illustrates the results of the meta-analysis in a forest plot.^6^

There was evidence of considerable heterogeneity: *Q*(108) = 433.17; *p* < 0.001. $I_{\text{Level 3}}^{2}=2.2\%$ of the total variation can be attributed to heterogeneity between studies, while $I_{\text{Level 2}}^{2}=80.9\%$ can be attributed to heterogeneity within studies.

### 3.2 PD studies

A recent methodological critique of using sacrificial dilemmas to test the mDPM points out that in sacrificial moral dilemmas, participants can only choose between two options (interve or do nothing). Therefore, studies that use these scenarios to test the mDPM need to assume that consequentialist and deontological responses are inversely related—when people endorse the consequentialist option in a sacrificial dilemma, they also reject (or would reject) the deontological option (and vice versa). However, deontology and consequentialism (the moral frameworks underlying these responses) are not related in this way, but are conceptually distinct. This is an issue for research on the mDPM, because even if it turned out that increased Type 2 processing results in more consequentialist responding and decreased Type 2 processing results in more deontological responding, this need not be evidence of two distinct types of cognitive processes, one consequentialist, one deontological. Instead, the same results are also consistent with single process accounts (e.g., Schein and Gray, 2018).

To do better, Conway and Gawronski (2013) have proposed to use *congruent sacrificial dilemmas* in addition to traditional sacrificial dilemmas. Like traditional dilemmas, congruent dilemmas pit a harmful action against a harmful inaction. However, in a congruent dilemma, the harm that would be caused if the agent intervened is greater than the harm that would result if the agent did nothing. The advantage of including both traditional and congruent dilemmas in a study is that its results can be analyzed using the *process dissociation* (PD) procedure (Jacoby et al., 1987). This procedure allows researchers to “independently quantify the strength of deontological and utilitarian inclinations” (Conway and Gawronski, 2013, p. 219). PD analysis outputs two quantities, *U* and *D*, where *U* measures the extent to which an individuals' moral judgments (about sacrificial dilemmas) are based on consequentialism, while *D* does the same for deontology. The DPM predicts that encouraging participants to engage in Type 2 processing will lead to higher values of *U*, while inhibiting Type 2 processing will lead to lower values of *U*. Since all PD studies also include traditional sacrificial dilemmas, my literature search should also have identified all published PD studies (within the parameters described in Figure 1). Nine articles, in addition to traditional sacrificial dilemmas, also used the PD analysis approach (*k* = 14). Here, I present meta-analytic results for 8 of these articles, reporting results from 12 individual studies [total *N* = 2, 577; *M*(*N*) = 214.8].^7^

**Figure 3:**
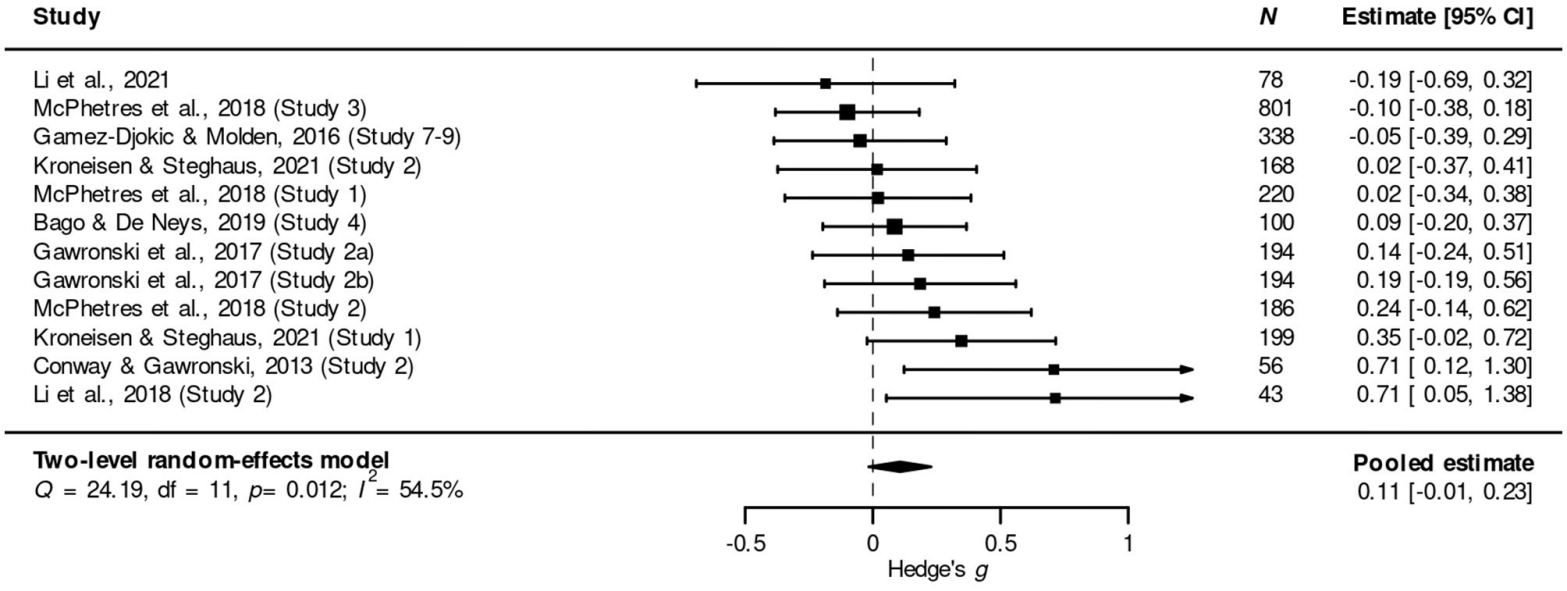
Forest plot showing the meta-analytic effect of four standard cognitive-processing manipulations on the PD parameter *U*. Positive values of *g* indicate results in line with the mDPM.

Again, I used Hedge's *g*, since the parameter *U* is continuous. *g* was once more coded so that positive values indicate results in line with mDPM: higher values of *U* for participants who were encouraged to engage in Type 2 processing compared to participants in a control condition or participants whose ability to engage in Type 2 processing was inhibited; lower values of *U* for participants whose ability to engage in Type 2 processing was inhibited compared to participants in a control condition. The pooled effect is *g* = 0.11, 95% CI = (−0.01, 0.23), *p* = 0.079. This effect is in the expected direction (from the perspective of the mDPM); however, it did not reach the conventional level of statistical significance (*p* < 0.05). Figure 3 illustrates the results in a forest plot.

### 3.3 Publication bias

*Publication bias* happens when the probability that a study gets published are affected by its results. Two common reasons for publication bias are the greater tendency of significant results (compared to non-significant results) to see the light of day and the greater tendency of results that support the initial hypothesis to get published (Fanelli, 2012; Ferguson and Brannick, 2012; Franco et al., 2014; Kühberger et al., 2014). Publication bias can make it difficult to draw valid meta-analytic conclusions; in particular, its presence often results in inflated overall effect size estimates, since studies that find weaker or conflicting evidence will tend to be missing from the literature (see Thornton and Lee, 2000; Rothstein et al., 2005).

One common method to attempt to detect publication bias uses *funnel plots* (Peters et al., 2008). In a funnel plot, effect size (*x*-axis) is plotted against the inverse of the standard error of the effect size (*y*-axis). If there is no publication bias, then this plot should look roughly like an upside-down funnel, since effect sizes with low standard errors (i.e., high precision) are expected to cluster near the pooled effect size, while effect sizes that are associated with higher standard errors (i.e., lower precision) will be more widely dispersed.

**Figure 4:**
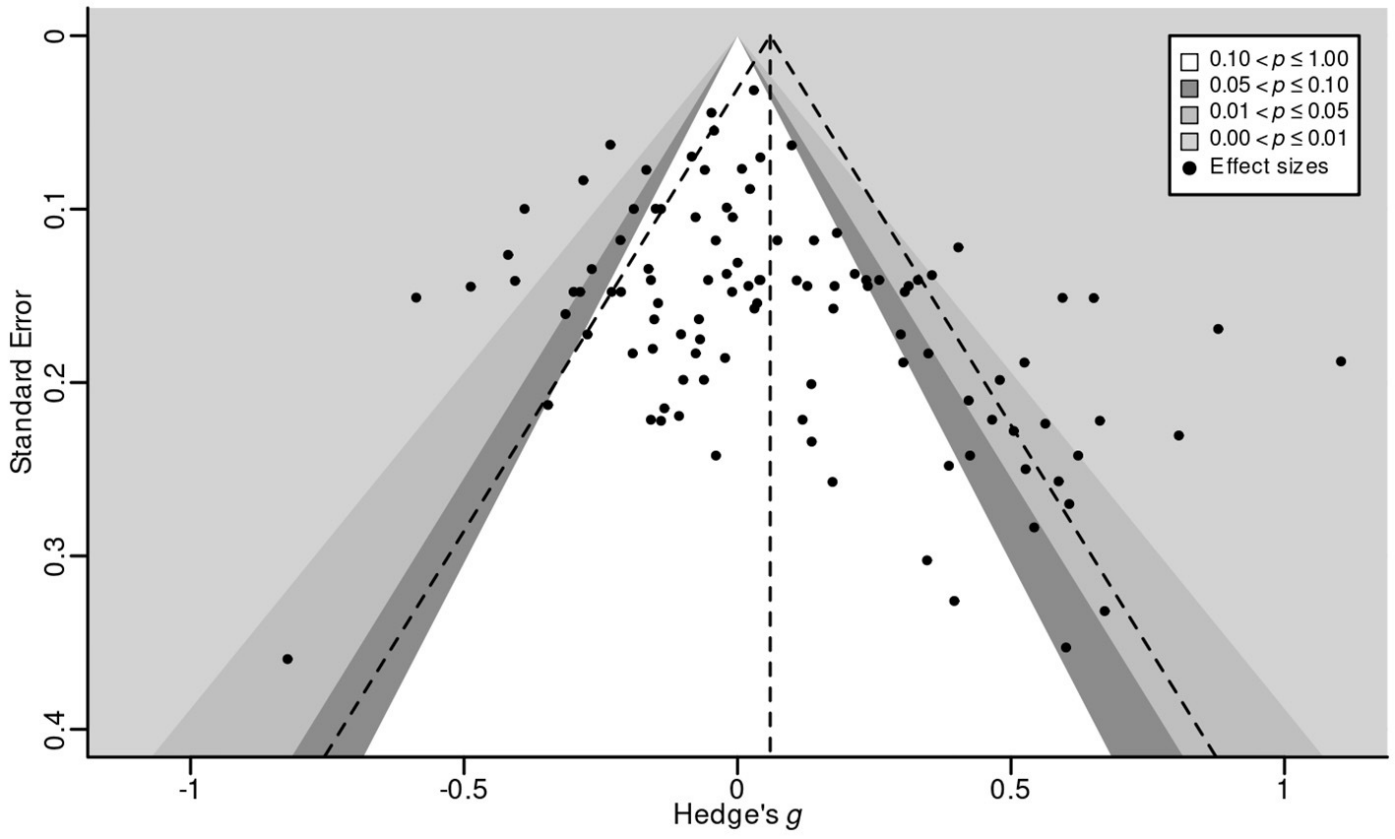
Contour-enhanced funnel plot of published effect sizes for the effect of four common cognitive-processing manipulations on moral judgments about acts or decisions in sacrificial moral dilemmas. Each dot represents a reported effect size; shaded regions show the level of statistical significance associated with effect sizes in that region. The vertical dashed line shows the pooled effect size; the two diagonal dashed lines indicate the boundaries of the pooled effect's 95% confidence region.

Figure 4 shows a contour-enhanced funnel plot for the current meta-analysis (excluding gray literature). Visual inspection suggests substantial asymmetry. More specifically, there are almost no effect sizes in the bottom-left area of the plot, meaning that there are few published studies with high standard errors that have reported negative effects. Supporting this impression, *Egger's regression test* (Egger et al., 1997) was significant, ${\hat{\beta }}_{0}=-0.17$, *t* = 3.22, *p* = 0.001. Both results strongly suggest (but do not prove; see Page et al., 2021b) the presence of small-*N* publication bias, in the sense that studies with high standard errors likely had a lower probability of getting published if their results contradicted the expectation of the mDPM.

One standard way to estimate the “true” effect size (that is, the effect size in the absence of publication bias) is Duval and Tweedie (2000)'s *trim-and-fill* method. The idea behind this method is to impute the studies that are assumed to be missing from the data set until the funnel plot is symmetric. The pooled effect size of this extended data set then represents the estimate corrected for small-study publication bias. When applied to the current set of studies (number of imputed studies = 17), the estimated effect of cognitive-processing manipulation on moral judgments of sacrificial dilemmas becomes negative, but remains statistically indistinguishable from zero, *g* = −0.02, 95% CI = (−0.09, 0.05), *p* = 0.488.

In addition, I also ran *PET-PEESE*, a more recent method to correct meta-analytic estimates for small-study publication bias (Stanley and Doucouliagos, 2014). The PET-PEESE-corrected estimate is significant, *g* = −0.11, 95% CI = (−0.20, −0.02), *p* = 0.007, but in the opposite direction from what the mDPM predicts. PET-PEESE is known to have a tendency to over-correct for bias, however, meaning that it may be more appropriate to interpret this result as suggesting the lack of a pooled effect instead of a negative pooled effect (Carter et al., 2019).

### 3.4 Meta-regression

**Figure 5:**
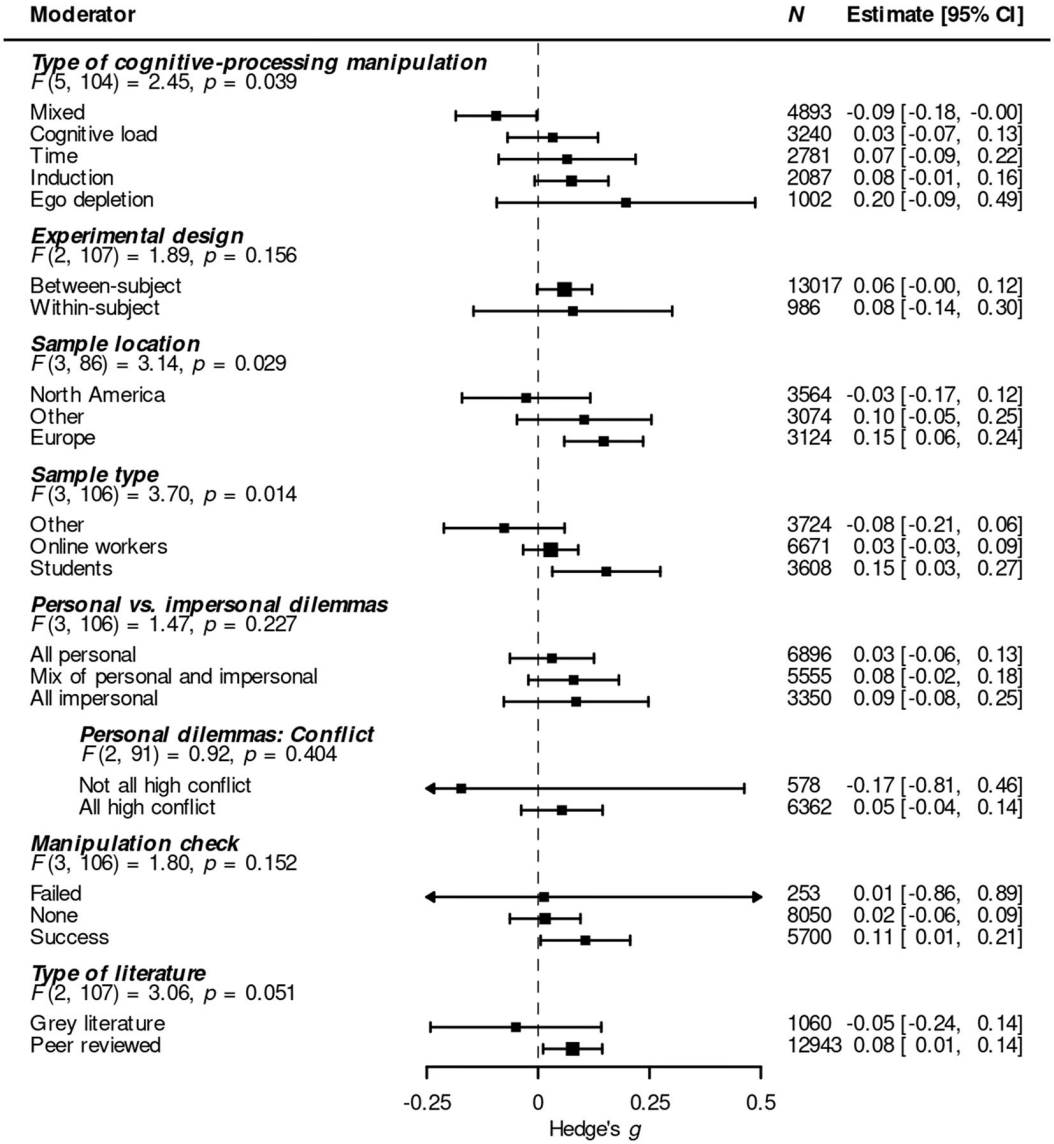
Forest plot illustrating the role of a series of potential moderators on the effect of four standard cognitive-processing manipulations on moral judgments about acts or decisions in sacrificial moral dilemmas. Positive values of *g* indicate results in line with the mDPM.

Next, I examined the role of a series of potential moderators: type of cognitive-processing manipulation (Horstmann et al., 2010; Isler and Yilmaz, 2023); experimental design (between-subject vs. within-subject; Morris and DeShon, 2002); geographic location of the sample (Henrich et al., 2010; Medin et al., 2017); participant pool (for example, students or online workers; Peterson, 2001; Stewart et al., 2017); type of sacrificial dilemma (impersonal or personal); the presence and status of a manipulation check; and type of literature (peer reviewed vs. gray). To this end, I used mixed-effects meta-regression, entering each potential moderator as a predictor into a three-level mixed-effects model (first level: individual effect sizes; second level: individual studies; third level: meta-analytic aggregate; see Harrer et al., 2021, sect. 10.1). Figure 5 illustrates the results in a forest plot.

#### 3.4.1 Type of cognitive-processing manipulation

Cognitive-processing manipulations likely differ in terms of their effectiveness (Horstmann et al., 2010; Isler and Yilmaz, 2023). For example, instead of inhibiting Type 2 processing, time pressure may in many cases reduce comprehension, making this manipulation less effective than other methods. Conversely, some induction manipulations (e.g., direct instructions) may be associated with considerable experimenter demand and so be more effective than other approaches.

Thirty-three effect sizes came from studies that used time restriction, followed by induction (*k* = 22), ego depletion (*k* = 17) and cognitive load (*k* = 21). Another 16 effect sizes had been found using a mix of more than one of the other four types (for details, see Table 1). Type of cognitive-processing manipulation had a significant overall effect on effect size: *F*_(5, 104)_ = 2.45, *p* = 0.039. The pooled effect of ego depletion studies was larger than that of the other manipulation types except for time restriction (*F*s≥4.03, *p*s ≤ 0.026). Moreover, the pooled effect of mixed studies was smaller than that of studies using ego depletion and induction (*F*s≥4.39, *p*s ≤ 0.02). It was also the only pooled effect statistically different from zero, though in the opposite direction of what the mDPM predicts: *g* = −0.09, 95% CI = (−0.18, 0.00), *p* = 0.043. None of the other pairwise differences reached statistical significance.

#### 3.4.2 Experimental design

Most effect sizes came from studies that used a between-subject design (*k* = 86; within-subject: *k* = 23). The effect of experimental design was not significant, *F*_(2, 107)_ = 1.89, *p* = 0.156.

#### 3.4.3 Sample location

As is common in social science research (Arnett, 2008; Rad et al., 2018; Thalmayer et al., 2021), a large majority of effect sizes came from samples of North Americans (*k* = 41) or Europeans (*k* = 35).^8^ In contrast, samples from other continents only contributed 13 effect sizes. Sample location had a significant on effect size, *F*_(3, 86)_ = 3.14, *p* = 0.029. The pooled effect of samples from Europe was significantly larger than the pooled effect of samples from Other or North America (*Fs*≥3.37; *ps* ≤ 0.04) and was the only pooled effect that reached statistical significance, *g* = 0.15, 95% CI = (0.06, 0.24), *p* = 0.002. No treatment of the mDPM that I am aware of discusses the potential role of sample location as a mediator, let alone predicts the specific differences just described.

#### 3.4.4 Sample type

Most effect sizes came from samples of online workers (from platforms like MTurk or Prolific; *k* = 37) or university students (*k* = 48; again, this is common for social science research: Arnett, 2008; Stewart et al., 2017; Thalmayer et al., 2021). Only 24 effect sizes were not from samples entirely made up of students or online workers. Type of subject pool had a significant overall effect on effect size, *F*_(3, 106)_ = 3.70, *p* = 0.014. More specifically, the pooled effect in student samples was larger than the pooled effect in samples from the other two types of subject pool (*F*s≥4.18, *p*s ≤ 0.019). The pooled effect for student samples was the only pooled effect that reached statistical significance [*g* = 0.15, 95% CI = (0.03, 0.27), *p* = 0.014]. No treatment of the mDPM that I am aware of discusses the potential role of sample type as a mediator, let alone predicts the specific differences found here.

#### 3.4.5 Personal vs. impersonal dilemmas

Since the mDPM was first introduced, there has been considerable disagreement regarding how best to use sacrificial moral dilemmas to test the model.^9^ Greene et al. (2001) distinguished between two types of sacrificial dilemmas, *personal* and *impersonal* dilemmas. In a personal dilemma, the agent's choice must involve serious bodily harm to one or more particular individuals, where this harm is not the result of deflecting an existing threat. Footbridge is a paradigmatic example of a personal sacrificial dilemma. Impersonal dilemmas fail to meet at least one of these conditions; one paradigmatic example is Switch. Greene et al. (2008, p. 1146) then recommended that only personal dilemmas be used to test the mDPM.

I assigned each effect size to one of three categories based on the proportion of personal dilemmas used in the study it originated from.^10^ Fifty-one effect sizes had been observed with sets of just personal dilemmas, while 16 effect sizes had been observed with sets of just impersonal dilemmas. The remaining effect sizes were based on a mix of personal and impersonal sacrificial dilemmas (*k* = 42). The overall effect of dilemma type was not significant: *F*_(3, 106)_ = 1.47, *p* = 0.227. Moreover, contrary to expectation, the pooled effect from sets of only personal dilemmas did not differ significantly from zero [*g* = 0.03, 95% CI = (−0.06, 0.13), *p* = 0.512] and was *smaller* than the pooled effect from sets of only impersonal dilemmas or from a mix of the two types (see Figure 5; neither difference was statistically significant).

#### 3.4.6 Person dilemmas: conflict

Koenigs et al. (2007) further split personal dilemmas into low conflict and high conflict dilemmas. In low conflict personal dilemmas, there is complete agreement or almost complete agreement among participants about which choice the agent should take. Conversely, in high conflict dilemmas, there is substantial disagreement among participants about which choice the agent should take. Greene et al. (2008), in addition to recommending that only personal dilemmas be used in tests of the mDPM, further stated that researchers should focus on high conflict dilemmas because “[o]nly high-conflict dilemmas are suitable for examining the conflict between utilitarian [= consequentialist] and non-utilitarian judgment processes” (p. 1148). A large majority (83.3%) of the effect sizes based on just personal dilemmas came from studies that used high conflict dilemmas exclusively.^11^ The pooled effect of studies with all high conflict personal dilemmas was not significantly different from zero, *g* = 0.05, 95% CI = (−0.04, 0.14), *p* = 0.244, and did not differ significantly from the pooled effect of studies which also featured some low conflict personal dilemmas: *F*_(2, 91)_ = 0.92, *p* = 0.404.

#### 3.4.7 Manipulation check

If a study fails to find an effect of a cognitive-processing manipulation on an outcome, one explanation is that cognitive processing does not have an impact on that particular outcome. An alternative explanation, however, is that the cognitive-processing manipulation in question failed to do what it was supposed to do. To help rule out this alternative explanation, manipulation checks are essential (see Horstmann et al., 2010, p. 228–230). Of the effect sizes included in the meta-analysis, 58 came from studies that included at least one manipulation check, while 51 came from studies that did not report any manipulation checks. The most common type of manipulation check compared participant response times across conditions (see Table 1), the thought being that participants who have been encouraged to rely on type 2 processing should take longer to respond than control participants or participants whose ability to engage in type 2 processing has been inhibited. Almost all manipulation checks were reported as successful (*k* = 56). The overall effect of manipulation check status was not significant: *F*_(3, 106)_ = 1.80, *p* = 0.152. In particular, the pooled effect of studies with successful manipulation checks did not differ significantly from the other two pooled effects (*F*s ≤ 2.68, *p*s≥0.074)—it did, however, differ significantly from zero, *g* = 0.11, 95% CI = (0.01, 0.21), *p* = 0.039.

#### 3.4.8 Type of literature

Ninety-seven effect sizes came from studies that had undergone peer review; the remaining 12 came from studies in the gray literature. The effect of type of literature was not significant, *F*_(2, 107)_ = 3.06, *p* = 0.051. However, it is worth noting that while the pooled effect of peer reviewed studies was significant and in the direction of the mDPM [*g* = 0.08, 95% CI = (0.01, 0.14), *p* = 0.023], the pooled effect of studies from the gray literature was in the opposite direct [though not significant; *g* = −0.05, 95% CI = (−0.24, 0.14), *p* = 0.578]. This further (see Section 3.3) suggest the presence of some amount of publication bias in the peer reviewed literature on the effect of cognitive-processing manipulations on moral judgments about acts or decisions in sacrificial moral dilemmas.

## 4 Discussion

Greene's influential dual-process model of moral cognition (mDPM; Greene et al., 2001; Greene, 2008, 2014) proposes that when people engage in Type 1 processing, they typically make deontological moral judgments, while when people engage in Type 2 processing, they typically make consequentialist moral judgments. Since the mDPM is a causal model, a convincing case for it requires experimental evidence. Historically, one major source of causal evidence that proponents of the mDPM have cited are studies that investigate the effect of experimental manipulations designed to either encourage or inhibit Type 2 processing on moral judgments about sacrificial moral dilemmas. This paper meta-analyzed published English-language studies with adult participants that used any of four standard types of cognitive-processing manipulations—cognitive load, ego depletion, induction and time restriction [*n* = 44; *k* = 68; total *N* = 14, 003; *M*(*N*) = 194.5]. The results do not support the mDPM. The overall pooled effect, while in the direction of the mDPM's prediction, was very small and did not differ significantly from zero [*g* = 0.06, 95% CI = (0.00, 0.12), *p* = 0.057]. A meta-analysis of a subset of studies [*n* = 8; *k* = 12; total *N* = 2, 577; *M*(*N*) = 214.8] that allowed for analysis using the process dissociation approach (Jacoby et al., 1987; Conway and Gawronski, 2013) also failed to find support for the mDPM.

One possible objection points out that many of effect sizes included in the first meta-analytic estimate came from studies that featured (some only featured) impersonal sacrificial moral dilemmas. However, Greene et al. (2008, p. 1147) argued that only personal sacrificial dilemmas should be used in tests of the mDPM, and so these results do not in fact threaten the mDPM. To address this response, I used meta-regression, but found that the pooled effect of studies that only included personal dilemmas did not differ significantly from zero or the pooled effect of studies that only used impersonal sacrificial dilemmas.

Another response is to argue that my results, instead of challenging the mDPM, speak only against the effectiveness of the cognitive-processing manipulations I included. If these manipulations had often been unsuccessful in encouraging or inhibiting Type 2 processing, then a significant pooled effect would not be expected even if the mDPM were true. However, I found no significant differences between effect sizes from studies with successful manipulation checks, effect sizes from studies with failed manipulation checks and effect sizes from studies where the authors did not report having used a manipulation check. Of course, manipulation checks can be inappropriate or insufficient, and so this result does not entirely rule out the possibility that most or even all studies used ineffective experimental manipulations. In that case, however, these studies would simply be uninformative and so would also not support the mDPM.

It may also be objected that I failed to include certain experimental manipulations and that, if these had been included, the results of the meta-analysis might have been more favorable to the mDPM. One such omission are studies that investigate the so-called foreign language effect. These studies compare moral judgments made in a foreign language with moral judgments made in one's native language (for a meta-analysis, see Circi et al., 2021). However, even though some authors have indeed suggested that this manipulation increases Type 2 processing and so can be used to test the mDPM (e.g., Brouwer, 2019; Circi et al., 2021), the existing evidence does not bear this out. Several studies have concluded that making moral judgments in a foreign language does not encourage Type 2 processing and instead point to increased psychological distance and reduced emotional responding as more likely explanations for the foreign language effect (e.g., Geipel et al., 2015; Corey et al., 2017; Hayakawa et al., 2017).

In their review of cognitive-processing manipulations, Horstmann et al. (2010) discuss another method I did not consider: manipulations of mood. The motivating idea behind these manipulations is that “sad mood leads people to analyze information more deliberately and thoroughly, whereas a happy mood activates more heuristic, intuitive strategies” (p. 227). At least two studies have investigated the effect of mood on moral judgments about sacrificial dilemmas (Valdesolo and DeSteno, 2006; Pastötter et al., 2013). While neither study unequivocally supports the mDPM^12^ and I know of no other studies like them, experimental manipulations of mood were outside the scope of my literature search.^13^ Therefore, for all I know, these studies are out there and their inclusion may have changed the results of the meta-analysis, perhaps to something more favorable to the mDPM. This is a clear limitation of my paper.

That said, defenders of the mDPM should not take too much comfort in this limitation. Recall that none of the four types of cognitive-processing manipulations I did include showed a significant individual meta-analytic effect. Moreover, the pooled effect of studies that used a combination of these types was significantly *smaller* than zero. This is notable, as Horstmann et al. (2010) recommend researchers use a combination of different cognitive-processing manipulations because “additive effects enhance the probability of a successful manipulation” (p. 233). If cognitive load, ego depletion, induction, time restriction and their combinations are typically successful at encouraging or inhibiting Type 2 processing, these results contradict the mDPM. The fact that I omitted experimental manipulations of mood can overturn this conclusion only if there is reason to think that these manipulations are superior to the cognitive-processing manipulations I did include. Otherwise, even if the meta-analytic effect of studies that manipulated mood had been significant and in the direction of the mDPM, the preponderance of the experimental evidence would still fail to support the model.

Yet another response is that the results of this meta-analysis do not threaten the mDPM because sacrificial moral dilemmas are a poor measure of consequentialist moral judgment (cf., Rosas and Koenigs, 2014; Kahane, 2015). Instead, we should look to research with alternative measures, for example, the Oxford Utilitarianism Scale (Kahane et al., 2018; Capraro et al., 2019). While I personally find this response compelling, for defenders of the mDPM, it comes at a very steep price. A large majority of the evidence for the mDPM—not just experimental evidence, but evidence of any kind—comes from studies with sacrificial moral dilemmas (see e.g., Greene, 2014, p. 700–705). Therefore, to admit that these scenarios are a poor measure of consequentialist moral judgment would be to dramatically undercut the model's empirical support. It is doubtful, then, that defenders of the mDPM would want to champion this particular objection.

A final response is to question the usefulness of meta-analyses, more generally. The results of a meta-analysis, so the objection, are only informative if the studies that go into it are of high quality (e.g., Egger et al., 2001; Borenstein et al., 2009). Conversely, if a “meta-analysis includes many low-quality studies, then fundamental errors in the primary studies will be carried over to the meta-analysis” (Borenstein et al., 2009, p. 380)—garbage in, garbage out. When it comes to the studies included in the present meta-analysis, there are indeed a few reasons for concern. Many of these studies featured relatively small samples and did not justify their sample size; almost half (43.5%) did not report having used a manipulation check; and very few studies had been pre-registered. Yet this objection (like the previous objection) again requires defenders of the mDPM to distance themselves from a substantial chunk of the evidence for their model. In other words: If the objection was successful, this victory would very much be a Pyrrhic victory.

## Data availability statement

Publicly available datasets were analyzed in this study. This data can be found here: https://osf.io/cnury/.

## Author contributions

PR: Conceptualization, Data curation, Formal analysis, Investigation, Methodology, Project administration, Software, Visualization, Writing – original draft, Writing – review & editing.

## Footnotes

^1^Short for moral dual-process model.

^2^Greene has also argued that deontological moral judgments (but not consequentialist moral judgments) are based on *emotional* processing. Note, however, that this claim is conceptually and empirically distinct from the claim that deontological moral judgments are typically the output of Type 1 processing (even though Greene does not always clearly distinguish the two claims; see Kahane, 2012, p. 522–3). In this paper, I am not concerned with the role of the emotions in deontological and consequentialist moral judgment.

^3^Recent reviews (Horstmann et al., 2010; Isler and Yilmaz, 2023) mention three other induction methods beyond explicit instruction and unconscious primes (enhancement of task relevance; monetary incentives; de-biasing training). However, I did not find any studies that employed any of these approaches and also matched the other inclusion criteria.

^4^The studies are Swann et al. (2014, Study 4), Kinnunen and Windmann (2013), Lane and Sulikowski (2017), and Vicario et al. (2018).

^5^I used the following packages: *dplyr* (Wickham et al., 2023a); *stringr* (Wickham, 2022); *metafor* (Viechtbauer, 2010); *esc* (Lüdecke, 2019); *bookdown* (Xie, 2023); *readr* (Wickham et al., 2023b), and *rmarkdown* (Allaire et al., 2023).

^6^Three studies (Killgore et al., 2007; Cummins and Cummins, 2012; Paxton et al., 2014) reported at least one large effect size (*g*>0.8; Cohen, 1988; recall that some studies reported more than one relevant effect size) in line with the predictions of the mDPM (see Figure 4). There were no obvious commonalities between these studies (for example, in terms of any of the attributes listed in Table 1).

^7^I was not able to include two studies (Białek and De Neys, 2017, Study 2 & 3). Neither study reports finding a significant effect.

^8^Sample location was not reported for 20 effect sizes; these effect sizes were excluded from this analysis (for details, see Table 1).

^9^Some researchers have argued that sacrificial dilemmas are very poor measures of deontological and consequentialist moral judgment, and so they should not be used to test the mDPM at all (e.g., Rosas and Koenigs, 2014; Kahane, 2015). While I find much of these critiques persuasive, many do not, and sacrificial moral dilemmas remain a common (perhaps the most common) measure of deontological and utilitarian moral judgment in moral psychological research.

^10^To do this, I first checked if the authors themselves stated the proportion of personal dilemmas in their study (with reference to Greene et al., 2001, where this the distinction between personal and impersonal dilemmas was first introduced). If that failed, I next checked if one or more of the dilemmas had been part of the dilemma battery used in Greene et al. (2001). If that too failed, I read the dilemma's text in full and then coded it myself based on the three criteria described in Greene et al. (2001).

^11^To determine this, I first checked if the authors themselves stated the proportion of high conflict personal dilemmas in their article (with reference to the distinction made in Koenigs et al., 2007). If that failed, I checked if one or more of the dilemmas had been part of the battery of high conflict personal dilemmas used in Koenigs et al. (2007). In this way, I was able to determine the proportion of high conflict personal dilemmas used in all but three reported effect sizes. Since the high conflict/low conflict distinction is an empirical distinction (unlike the personal/impersonal distinction, which is a conceptual distinction), I was not able to categorize these remaining instances myself and so they were excluded from the analysis.

^12^Valdesolo and DeSteno (2006) found that positive mood increased, rather than decreased, consequentialist responses in Footbridge; Pastötter et al. (2013) found that the effects of positive and negative mood on moral judgments in Footbridge depends on decision frame (active vs. passive).

^13^Greene has also argued that deontological (but not consequentialist) moral judgments are based on emotional processes (see text footnote^1^). While logically distinct from the mDPM's claim that deontological (but not consequentialist) moral judgments are typically the output of Type 1 processing, the two claims are not always clearly disentangled. Studies that investigate the effect of mood on moral judgments about sacrificial dilemmas bear not only on the mDPM, but also on this emotions claim—indeed, this is the context in which Valdesolo and DeSteno (2006) and Pastötter et al. (2013) manipulated mood. As the causal role of the emotions was not the topic of this meta-analysis, I chose to exclude mood manipulations, to avoid an interpretational muddle.
